# Plasma Prolactin and Progesterone Levels and the Risk of Gestational Diabetes: A Prospective and Longitudinal Study in a Multiracial Cohort

**DOI:** 10.3389/fendo.2020.00083

**Published:** 2020-02-27

**Authors:** Mengying Li, Yiqing Song, Shristi Rawal, Stefanie N. Hinkle, Yeyi Zhu, Fasil Tekola-Ayele, Assiamira Ferrara, Michael Y. Tsai, Cuilin Zhang

**Affiliations:** ^1^Epidemiology Branch, Division of Intramural Population Health Research, Eunice Kennedy Shriver National Institute of Child Health and Human Development, Bethesda, MD, United States; ^2^Epidemiology Department, Richard M. Fairbanks School of Public Health, Indiana University, Indianapolis, IN, United States; ^3^Department of Nutritional Sciences, School of Health Professions, Rutgers University, Newark, NJ, United States; ^4^Division of Research, Kaiser Permanente Northern California, Oakland, CA, United States; ^5^Department of Epidemiology & Biostatistics, University of California, San Francisco, San Francisco, CA, United States; ^6^Department of Laboratory Medicine and Pathology, University of Minnesota Medical School, Minneapolis, MN, United States

**Keywords:** prolactin, progesterone, gestational diabetes, glucose homeostasis biomarkers, longitudinal studies

## Abstract

**Objective:** Prolactin and progesterone are implicated in glucose homeostasis in and outside of pregnancy. However, their associations with gestational diabetes (GDM) risk were not well-understood. This study investigates this question in a prospective and longitudinal cohort.

**Methods:** This is a nested case-control study of 107 incident GDM cases and 214 matched non-GDM controls within the NICHD Fetal Growth Studies-Singleton Cohort. Blood samples were collected at gestational weeks 10–14, 15–26, 23–31, and 33–39. The odds ratios (OR) of GDM were estimated using conditional logistic regression. The longitudinal changes in prolactin and progesterone were estimated using linear mixed-effects models.

**Results:** Compared to controls, cases have significantly higher prolactin levels at weeks 10–14 (median: 50.4 vs. 42.1 ng/mL), and significantly lower progesterone levels at weeks 10–14 (median: 109.4 vs. 126.5 nmol/L). Prolactin levels at weeks 10–14 were significantly and positively associated with GDM risk; the adjusted ORs across increasing quartiles were 1.00, 1.13, 1.80, 2.33 (*p*-trend = 0.02). A similar but slightly attenuated association was observed at weeks 15–26 (*p*-trend = 0.05). Progesterone was not associated with GDM risk at either time points. Longitudinal changes in prolactin and progesterone between the first two visits were not associated with GDM risk. In addition, prolactin was significantly and positively associated with insulin and C-peptide levels at weeks 10–14, and significantly and inversely associated with C-peptide levels at weeks 15–26; progesterone was significantly and inversely associated with glucose and insulin levels.

**Conclusions:** This study provided the first prospective evidence of a positive association between prolactin levels in early pregnancy and GDM risk.

## Introduction

Normal pregnancy is characterized by a progressive decline in insulin sensitivity starting around mid-pregnancy accompanied by a compensatory increase in insulin secretion (1). During pregnancy, several reproductive hormones, including prolactin and progesterone, rise continuously and reach many folds of their pre-pregnant levels (2), and these hormones are long thought to play a central role in regulating the metabolic changes in pregnancy (3, 4). Gestational diabetes is glucose intolerance resulted from insufficient insulin supply relative to the degree of insulin resistance in pregnancy. Understanding the potential roles of prolactin and progesterone in the development of GDM could help to reveal the pathophysiology of GDM.

Prolactin is a pituitary hormonal primarily known for its role in lactation. However, it is also a key metabolic hormone implicated in multiple functions, including the regulation of body weight and appetite, adipose tissue function, and β-cell proliferation and insulin secretion (5). Interestingly, the overall effects of prolactin on metabolic health and glucose homeostasis might vary depending on its circulatory concentrations, as suggested by epidemiologic evidence. For example, elevated prolactin levels above the normal range (i.e., hyperprolactinemia) such as in the case of prolactinoma have been associated with adverse outcomes including hyperinsulinemia (6), insulin resistance (6–8), and increased body weight (9, 10). In contrast, higher prolactin levels within the normal range have been linked to lower risks of diabetes in middle-aged, non-pregnant populations (11, 12). As prolactin levels increase well-beyond the normal non-pregnant levels during pregnancy, higher prolactin levels during pregnancy might contribute to worsening glucose homeostasis. Progesterone is a steroid hormone produced first by corpus luteum and then by the placenta during pregnancy. It is responsible for maintaining pregnancy (13). Animal studies have reported conflicting findings regarding the role of progesterone in regulating β-cell proliferation and insulin secretion, with some suggesting a stimulatory role (14, 15) while others suggesting an inhibitory one (16, 17).

Although prolactin and progesterone have been implicated in regulating glucose metabolism, their associations with GDM risk has been inadequately investigated. Most studies of the associations of prolactin and progesterone with GDM risk are cross-sectional or retrospective in design (18–24). As the hormonal levels were measured at the time of or after GDM screening and diagnosis (typically around 26–28 weeks), these studies missed the early part of pregnancy preceding the clinical onset of GDM, a time when the pathophysiology of GDM already starts (1). Only one prospective study with five GDM cases (25) exists so far, which found no significant association between progesterone and prolactin levels and GDM risk in early or late pregnancy. Interpretations of this finding is hindered by limited statistical power.

In a case-control study nested within a large pregnancy cohort, we investigate the prospective and longitudinal associations of prolactin and progesterone levels and changes in these levels during pregnancy with subsequent GDM risk. We also explore the associations of these hormones with glucose homeostasis biomarkers prior to the screening and diagnosis of GDM.

## Materials and Methods

### Study Population

The *Eunice Kennedy Shriver* National Institute of Child Health and Human Development (NICHD) Fetal Growth Studies-Singletons is a multicenter, multiracial prospective pregnancy cohort. The cohort included 2,334 non-obese (BMI < 30 kg/m^2^) and 468 obese (BMI ≥ 30 kg/m^2^) women aged 18–40 years with singleton pregnancies enrolled between 8 and 13 weeks of gestation from 2009 to 2013. Women were excluded if they had pre-exidsting diabetes, hypertension, or other major chronic conditions (i.e., asthma, autoimmune disorders, cancer, chronic renal disease, epilepsy or seizure, hematological disorders, HIV or AIDs, psychiatric disorders, and thyroid disease). Furthermore, non-obese women were excluded if they had lifestyle risk-factors (i.e., used illicit drugs in the past year, smoked in the past 6 months, or consumed at least one alcoholic drink per day in pregnancy), had a history of obstetric complications, conceived using assisted reproductive technology. Research approval was obtained from all participating institutions and the participants provided written informed consent prior to their inclusion in the study.

Within the NICHD Fetal Growth Studies-Singleton Cohort, we identified 107 incident GDM cases via medical record review, defined according to the Carpenter-Coustan criteria (26) (i.e., at least two of the plasma glucose levels are met or exceeded: fasting−95 mg/dL, 1 h−180 mg/dL, 2 h−155 mg/dL, 3 h−140 mg/dL), and/or by receipt of GDM medications. We randomly selected 214 non-GDM controls individually matched to the cases in a 2:1 ratio based on age (±2 years), race/ethnicity (non-Hispanic white, non-Hispanic black, Hispanic, or Asian/Pacific Islander), and the gestational week of blood collection (± 2 weeks). As a result, a total of 321 women were included in this study. The matching design improves the efficiency of the study to address confounding from the matching variables.

### Blood Collection and Laboratory Tests

Following a standardized protocol, blood specimens were collected at four study visits. The visits were targeted at gestational weeks 8–13, 16–22, 24–29, and 34–37, but the actual ranges were weeks 10–14 (visit 1), 15–26 (visit 2), 23–31 (visit 3), and 33–39 (visit 4), respectively. The specimens at 15–26 weeks were collected after an overnight fast. All biospecimens were immediately processed and stored at −80°C until thawed for laboratory analysis. For the two study visits before GDM screening (i.e., weeks 10–14 and 15–26), biomarkers were measured in all cases and the two matched controls; for the two visits after GDM screening (i.e., weeks 23–31 and 33–39), biomarkers were measured in all cases and one of the two matched controls. Prolactin and progesterone were measured in plasma using a quantitative sandwich enzyme immunoassay (R&D Systems, Inc., Minneapolis, MN) and a competitive immunoassay method (Roche Diagnostics, Indianapolis, IN 46250), respectively. Glucose, insulin, and C-peptide were measured in plasma using hexokinase assay, immunosorbent assay, sandwich immunoassay (Roche Diagnostics, Indianapolis, IN), respectively. HbA1c was measured in whole blood using a non-porous ion-exchange high-performance liquid chromatography assay (Tosoh Automated Analyzer HLC-723G8, Tosoh Bioscience, Inc., South San Francisco, CA & Tokyo, Japan). The assay coefficient of variation (CV) was <1.16%. All other assays had CVs <10% and were performed without knowledge of GDM status. Homeostasis model assessment of insulin resistance (HOMA-IR) was calculated in the fasting sample collected at 16–22 works using the formula: HOMA-IR = (glucose [mg/dL] × insulin [μU/mL])/405 (27).

### Covariates

At the enrollment visit (gestational weeks 10–14), women reported their age, race/ethnicity, level of education, marital status, parity, and family history of diabetes in a structured questionnaire. Women in the obese cohort also reported smoking during the 6 months prior to pregnancy and current alcohol use. Pre-pregnancy body mass index (BMI) was calculated from self-reported pre-pregnancy weight and height measured at enrollment. Self-reported weight was highly correlated with weight measured by study personnel during the enrollment visit (r = 0.97). The gestational week at each visit was calculated from the last menstrual period.

### Statistical Analysis

Descriptive statistics were presented for the characteristics of cases and controls. Continuous variables were compared between cases and controls using linear mixed-effects models with a random intercept to account for the matched case-control design; categorical variables were compared between cases and controls using logistic regression models with generalized estimating equations.

The median values of prolactin and progesterone at each study were plotted in cases and controls separately to demonstrate their longitudinal trajectories over pregnancy; median instead of mean values were plotted to accommodate the non-normal distribution of the hormones. To compare the hormonal levels between cases and controls, we first tested the overall difference in hormonal levels across all four visits between cases and controls using linear mixed-effects models (dependent variable: hormonal levels; independent variable: case-control status); the model included a random intercept for groups of one case and two matched controls to account for the correlation within each group. If the difference was significant, we then tested the difference in hormonal levels at each study visit using a similar model. The hormonal levels were log-transformed before fitting the models to achieve normal distribution.

To examine the prospective associations of the hormonal levels with GDM risk, we estimated the odds ratios (ORs) and 95% confidence intervals (CIs) of GDM by quartiles of hormonal levels using conditional logistic regression models, consistent with the matched case-control design. We first fitted the crude model without adjusting for covariates, then we fitted the adjusted model including selected major risk factors of GDM as covariates, i.e., maternal age (years), gestational age at blood collection (weeks), pre-pregnancy BMI (kg/m^2^), and family history of diabetes (yes, no). Maternal age and gestational age at blood collection were included as covariates to control for residual confounding, as matching on these variables were not exact, but based on intervals. Smoking in the 6 months prior to pregnancy was not included as a covariate, as only five obese women reported such occurrence (non-obese women with such occurrence were ineligible for the study). The linear trend of the association across increasing quartiles of the hormonal levels was tested using the median value of each quartile as a continuous variable. The OR per unit increase in the hormone was estimated using the hormonal levels as a continuous variable. Only hormonal levels at the two visits prior to GDM screening (weeks 10–14 and 15–26) were evaluated in relation to GDM risk to follow a prospective design. To evaluated potential confounding from other biomarkers identified to be associated with GDM risks in our previous works (i.e., insulin-like growth factors [IGF], thyroid function markers, iron status biomarkers) (28–30), we examine the associations of prolactin and progesterone with these biomarkers, and adjusted for any biomarkers associated with prolactin or prolactin in the main analysis as a sensitivity analysis. We also evaluated potential effect modifications by stratifying the main analyses by pre-pregnancy BMI (<25, 25–29, and ≥30 kg/m^2^), parity (nulliparous, parous), family history of diabetes (yes, no), and infant sex (female, male).

To examine longitudinal changes in the hormones in relation to GDM risk, we modeled the hormonal levels at the two visits prior to GDM screening (weeks 10–14 and 15–26) as a function of case status (case, control) and study visit (weeks 10–14 and 15–26) using linear mixed-effects models with a random intercept to account for the matched case-control design; an interaction term between case status and study visit was included to estimate the difference in longitudinal changes of the hormone between cases and controls. Both the crude model and the adjusted model including selected major risk factors of GDM were fitted. The hormonal levels were log-transformed to achieve normal distributions before fitting the models. The least-square means estimated from the adjusted models were back-transformed to the original scale and plotted in a figure.

Lastly, to explore potential mechanisms linking the hormonal levels with GDM risk, we examined the associations of the hormonal levels with glucose hemostasis markers using Pearson's partial correlation adjusting for selected major risk factors of GDM. The biomarkers including glucose, insulin, C-peptide, and HbA1c were measured using blood samples collected weeks 10–14 and 15–26 and HOMA-IR at weeks 15–26. To reflect the associations in the general population, the cases and the controls were pooled, and the sample was weighted by the inverse probability of selection from the full cohort.

Throughout the analysis, to ensure the integrity of the prospective study design at weeks 10–14 and 15–26, we excluded one case at weeks 10–14 and five cases at weeks 15–26 who had blood samples collected after the diagnosis of GDM. A complete case analysis was used. All analyses were conducted using SAS version 9.4 (SAS Institute, Cary, NC).

## Results

**Figure 1:**
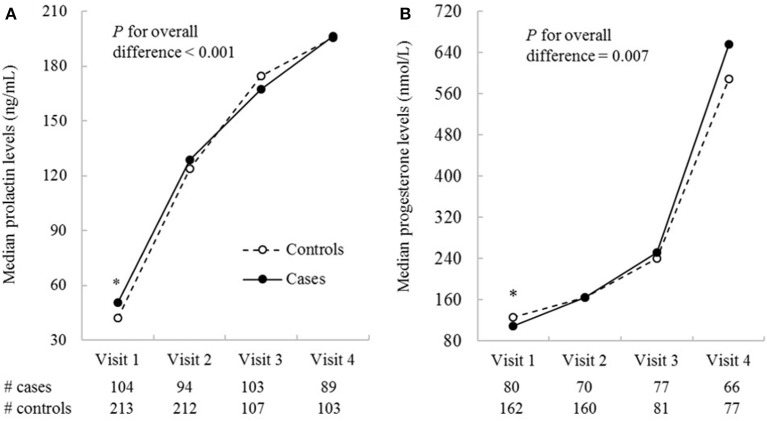
Median prolactin **(A)** and progesterone **(B)** concentrations by study visits in GDM cases and non-GDM controls in the NICHD Fetal Growth Studies-Singleton Cohort. Biomarkers were measured in all cases and the two matched controls for visit 1 and 2 (the two study visits before GDM screening), and in all cases and one of the two matched controls for visit 3 and 4 (the two visits after GDM screening). The four visits correspond to weeks 10–14, 15–26, 23–31, and 33–39. *P ≤ 0.05 for differences in log-transformed hormonal levels between GDM cases and non-GDM controls obtained from linear mixed-effects models accounting for matched case-control pairs at a specific study visit.

Compared to controls, the GDM cases were more likely to have a family history of diabetes and a higher pre-pregnancy BMI (Table 1). The trajectories of prolactin and progesterone levels increased progressively over pregnancy in both cases and controls (Figure 1). Overall, prolactin (*p* for overall difference <0.001) and progesterone (*p* for overall difference = 0.007) levels were both significantly different between cases and controls over the four study visits. Specifically, prolactin levels were significantly higher in cases than controls at weeks 10–14, the median [interquartile range (IQR)] were 50.4 (35.1, 74.5) vs. 42.1 (30.0, 66.3) ng/mL (*p* = 0.02); at weeks 15–26, the difference became smaller and short of significance (*p* = 0.05). Prolactin levels did not differ significantly between cases and controls at the two subsequent visits. Progesterone levels were significantly lower in cases than controls at weeks 10–14; the median (IQR) were 109.4 (88.6, 146.4) vs. 126.5 (97.9, 149.9) nmol/L, *p* = 0.01). Progesterone levels did not differ significantly between cases and controls at the three subsequent visits. The hormonal levels by status of selected covariates were shown in Supplementary Table 1.

**Table 1:** Background characteristics of GDM cases and non-GDM control in the NICHD Fetal Growth Studies-Singleton Cohort.

	**GDM cases** **(*n* = 107)**	**Non-GDM controls** **(*n* = 214)**	***P-*value^a^**
Age^b^, years, mean ± SD	30.5 ± 5.7	30.4 ± 5.4	
Race/ethnicity^b^, *n* (%)			
Non-Hispanic white	25 (23.4)	50 (23.4)	
Non-Hispanic black	15 (14.0)	30 (14.0)	
Hispanic	41 (38.3)	82 (38.3)	
Asian/Pacific Islander	26 (24.3)	52 (24.3)	
Education, *n* (%)			0.18
Less than high-school	17 (15.9)	26 (12.1)	
High-school graduate or equivalent	15 (14.0)	23 (10.7)	
More than high-school	75 (70.1)	165 (77.1)	
Married/living with a partner, *n* (%)	92 (86.0)	167 (78.0)	0.12
Nulliparous, *n* (%)	48 (44.9)	96 (44.9)	1.00
Family history of diabetes, *n* (%)	40 (37.4)	48 (22.4)	0.005
Pre-pregnancy BMI, *n* (%)			<0.001
<25.0 kg/m^2^	37 (34.6)	125 (58.4)	
25.0–29.9 kg/m^2^	35 (32.7)	56 (26.2)	
≥30.0 kg/m^2^	35 (32.7)	33 (15.4)	

a *P-values for differences between cases and controls were obtained by linear mixed-effects models for continuous variables and binomial/multinomial logistic regression with generalized estimating equations for binary/multilevel categorical variables, accounting for matched case-control pairs. Differences in matching variables (age and race/ethnicity) between cases and controls cannot be tested*.

b *Matching variables*. *SD, standard deviation*.

Higher prolactin levels were generally associated with a higher risk of GDM. At weeks 10–14, the adjusted ORs (95% CI) of GDM across increasing quartiles of prolactin levels were 1.00, 1.13 (0.52, 2.42), 1.80 (0.85, 3.80), and 2.33 (1.09, 4.99) (*p*-trend = 0.02); each 10 ng/mL increase of prolactin was associated with an OR of 1.13 (95% CI [1.03, 1.25], *p* = 0.02). Similar but slightly attenuated associations were observed at weeks 15–26 (*p*-trend = 0.05); each 10 ng/mL increase of prolactin was associated with an OR of 1.08 (95% CI [1.02, 1.14], *p* = 0.01). Higher progesterone levels at weeks 10–14 were associated with a lower risk of GDM which became non-significant after adjusting for covariates (*p*-trend = 0.10). Progesterone levels at weeks 15–26 were not associated with GDM risk (Table 2). Prolactin was not significantly associated with most biomarkers identified to be associated with GDM risks in our previous works (i.e., insulin-like growth factors, thyroid function markers, iron status biomarkers) (28–30). The main findings did not alter and mostly remained significant after adjusting for these biomarkers (Supplementary Table 2). In the stratified analyses, the observed associations were not modified by pre-pregnancy BMI, parity, family history of diabetes, or infant sex (data not shown).

**Table 2:** Odds ratios (95% confidence intervals) of GDM by prolactin and progesterone levels at gestational weeks 10–14 and 15–26 in the NICHD Fetal Growth Studies-Singleton Cohort.

	**GDM cases, *n***	**Non-GDM controls, *n***	**Crude**	**Adjusted^a^**
			**OR (95% CI)**	**OR (95% CI)**
**GESTATIONAL WEEKS 10–14**				
Prolactin, ng/mL				
1st quartile: 0.2–30.0	21	54	1.00	1.00
2nd quartile: 30.5–42.1	18	50	0.95 (0.47, 1.94)	1.13 (0.52, 2.42)
3rd quartile: 42.6–66.3	32	51	1.68 (0.85, 3.31)	1.80 (0.85, 3.80)
4th quartile: 66.8–184.2	33	52	1.75 (0.88, 3.47)	2.33 (1.09, 4.99)
*P*-trend	104	207	0.06	0.02
Per 10 ng/mL	104	207	1.10 (1.01, 1.20)	1.13 (1.03, 1.25)
Progesterone, nmol/L				
1st quartile: 48.0–97.9	33	41	1.00	1.00
2nd quartile: 100.8–125.8	19	39	0.61 (0.30, 1.22)	0.67 (0.31, 1.44)
3rd quartile: 127.1–149.9	13	41	0.37 (0.17, 0.84)	0.44 (0.18, 1.23)
4th quartile: 150.0–256.5	15	39	0.44 (0.20, 0.98)	0.52 (0.21, 1.28)
*P*-trend	80	160	0.01	0.10
Per 10 nmol/L	80	160	0.93 (0.86, 1.01)	0.97 (0.88, 1.06)
**GESTATIONAL WEEKS 15–26**				
Prolactin, ng/mL				
1st quartile: 0.1–84.3	19	48	1.00	1.00
2nd quartile: 84.7–123.2	21	49	1.13 (0.54, 2.37)	0.96 (0.42, 2.21)
3rd quartile: 124.4–161.3	27	44	1.66 (0.78, 3.55)	1.88 (0.82, 4.31)
4th quartile: 161.6–363.8	27	46	1.67 (0.74, 3.77)	2.12 (0.86, 5.19)
*P*-trend	94	187	0.16	0.05
Per 10 ng/mL	94	187	1.06 (1.01, 1.11)	1.08 (1.02, 1.14)
Progesterone, nmol/L				
1st quartile: 66.5–144.8	26	32	1.00	1.00
2nd quartile: 145.2–164.7	9	37	0.30 (0.12, 0.75)	0.49 (0.18, 1.33)
3rd quartile: 164.9–185.9	15	35	0.56 (0.25, 1.27)	0.87 (0.34, 2.21)
4th quartile: 186.3–362.5	20	35	0.78 (0.35, 1.77)	1.44 (0.53, 3.95)
*P*-trend	70	139	0.59	0.34
Per 10 nmol/L	70	139	0.99 (0.92, 1.06)	1.06 (0.97, 1.15)

a *Adjusted for maternal age (years), gestational age (weeks) at blood collection, pre-pregnancy BMI (kg/m^2^) and family history of diabetes (yes, no)*.

**Figure 2:**
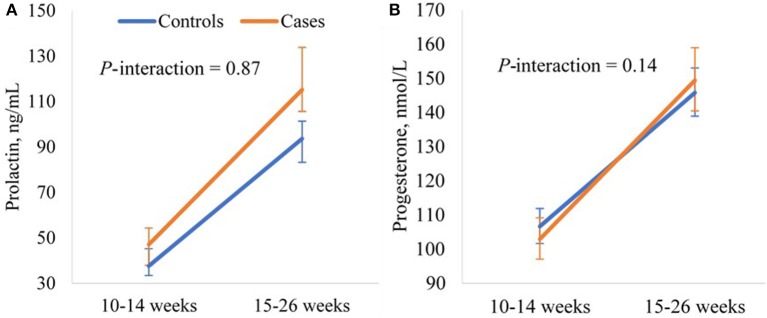
Adjusted longitudinal trajectories of **(A)** prolactin and **(B)** progesterone levels from gestational weeks 10–14 to 15–26 for GDM cases and non-GDM controls in the NICHD Fetal Growth Studies-Singleton Cohort. Least-square means and 95% confidence intervals estimated from the linear mixed-effects models adjusting for maternal age (years), pre-pregnancy BMI (kg/m^2^), and family history of diabetes (yes, no). Prolactin and progesterone levels were log-transformed before fitting the model and least-square means were back-transformed.

In the linear mixed-effects models examining longitudinal changes in the hormonal levels from weeks 10–14 to 15–26, GDM was associated with significantly higher prolactin levels at weeks 10–14 (*p* = 0.01), but not the magnitude of change in prolactin levels from weeks 10–14 to 15–26 (*p*-interaction = 0.87). GDM was not associated with progesterone levels at weeks 10–14 (*p* = 0.33). While GDM appeared to be associated with a larger increase in progesterone levels from weeks 10–14 to 15–26, the association was not significant (*p*-interaction = 0.14) (Supplementary Table 3). The adjusted least-square means illustrated significantly higher prolactin levels in cases than controls at both weeks 10–14 (mean 47.1 vs. 37.6 ng/mL, *p* = 0.01) and 15–26 (mean 115.2 vs. 93.7 ng/mL, *p* = 0.02), and similar progesterone levels in cases and controls at both visits (Figure 2).

Associations of prolactin and progesterone levels with glucose homeostasis biomarkers varied by gestational age. At weeks 10–14, prolactin levels were significantly and positively associated with insulin (*r* = 0.25, *p* < 0.001) and C-peptide levels (*r* = 0.23, *p* < 0.001). However, at gestational weeks 15–26, prolactin levels became not associated with insulin levels and inversely associated with C-peptide levels (*r* = −0.12, *p* = 0.04). Progesterone levels at gestational weeks 10–14 were significantly and inversely associated with glucose (*r* = −0.23, *p* < 0.001), insulin (*r* = −0.28, *p* < 0.001), and C-peptide levels (*r* = −0.33, *p* < 0.001). However, at weeks 15–26, progesterone levels became not associated with the biomarkers (Table 3).

**Table 3:** Pearson's partial correlation coefficients (*r*) of prolactin and progesterone with biomarkers of glucose and insulin homeostasis at weeks 10–14 and 15–26 among the combined sample of GDM cases and non-GDM controls^a^ in the NICHD Fetal Growth Studies-Singleton Cohort.

	**Gestational weeks 10–14^b^**				**Gestational weeks 15–26^c^**			
	**Prolactin**		**Progesterone**		**Prolactin**		**Progesterone**	
	***r^d^***	***P*-value**	***r^d^***	***P*-value**	***r^***d***^***	***P*-value**	***r^d^***	***P*-value**
Glucose	0.07	0.20	−0.23	<0.001	−0.02	0.71	−0.10	0.14
Insulin	0.25	<0.001	−0.28	<0.001	−0.08	0.15	−0.06	0.39
C-peptide	0.23	<0.001	−0.33	<0.001	−0.12	0.04	−0.08	0.25
HOMA-IR	–	–	–	–	−0.08	0.19	−0.05	0.49
HbA1c, %	0.03	0.66	−0.11	0.08	0.03	0.66	−0.01	0.84

a *The sample was weighted by the inverse probability of selection to represent the entire study cohort*.

b *Non-fasting sample*.

c *Fasting sample*.

d *Adjusted for maternal age (years), gestational age (weeks), pre-pregnancy BMI (kg/m^2^), and family history of diabetes (yes, no)*. *GDM, gestational diabetes; BMI, body mass index; HOMA-IR, homeostatic model assessment of insulin resistance*.

## Discussion

In this case-control study of GDM nested within a large pregnancy cohort, we found the first prospective evidence of a significant and positive association of early pregnancy prolactin levels with subsequent risk of GDM. We did not find evidence of an association between progesterone levels and subsequent risk of GDM.

Existing studies on the association between prolactin levels and GDM risk are mostly cross-sectional or retrospective (18–24); these studies did not capture the early part of pregnancy before GDM screening and diagnosis, which may be important for the development of GDM (1). To our knowledge, only one prospective study exists (25), with one prolactin measure before GDM diagnosis. It found the prolactin levels at weeks 12–14 to be numerically higher among five obese women with GDM than four obese women without GDM, but the difference was not significant (25). Interpretations of the finding is hindered by limited statistical power. Interestingly, one study found prolactin measured around week 30 to be significantly and inversely related to postpartum prediabetes/diabetes (31), but the same study found this prolactin measure to be not associated with GDM risk (23). The null association between a late pregnancy prolactin measure and GDM risk is consistent with our findings at weeks 23–31.

Within 107 GDM cases, our study is the largest prospective study so far, with longitudinal prolactin measures. We found that in early pregnancy at weeks 10–14, prolactin levels were higher in cases than controls, and that prolactin levels were significantly and positively associated with GDM risk after adjusting for selected major risk factors of GDM, including pre-pregnancy BMI; the findings were slightly attenuated for weeks 15–26 and were short of significance. As lower prolactin levels in pregnancy were associated with overweight before pregnancy (22), adjusting for pre-pregnancy BMI avoided potential confounding from it. However, we did not observe a difference in changes of prolactin levels from week 10–14 to 15–26 between cases and controls; instead, prolactin levels increased similarly in cases and controls from weeks 10–14 to 15–26, and the adjusted mean square estimates of prolactin levels were higher in cases than control at both time points.

Our finding of a positive association between prolactin levels and GDM risk in pregnancy is in line with findings among non-pregnant populations with prolactinoma, where elevated prolactin levels above the normal range (i.e., hyperprolactinemia) were associated with adverse metabolic outcomes including hyperinsulinemia (6), insulin resistance (6–8), and increased body weight (9, 10), and normalizing prolactin levels reverted the adverse metabolic outcomes (8–10); these findings support a role of hyperprolactinemia in worsening metabolic outcomes. In contrast, among non-pregnant, middle-aged populations, higher prolactin levels within the normal range (i.e., 2–18 ng/mL for men and 2–29 ng/mL for non-pregnant women) has been prospectively associated with lower risks of diabetes (11, 12) and impaired glucose regulation (11). The seemingly contradictory findings might be explained by potentially dose-dependent effects of prolactin on glucose homeostasis, as observed in an animal study, where a modest increase (2.5 folds) in prolactin levels promoted glucose-stimulated insulin secretion and reduced insulin resistance, but a large increase (11.8 folds) worsened insulin resistance, although both conditions stimulated β-cell expansion (32). Indeed, prolactin levels were much lower in the studies among non-pregnant, middle-aged individuals (median prolactin levels were 8–11 ng/mL) (11, 12) than in the studies among hyperprolactinemia patients (median prolactin levels ranges between 59 and 3,354 ng/mL) and in our study (median prolactin levels were 42 and 124 ng/mL at gestational weeks 10–14 and 15–26, respectively).

Prolactin has been implicated in multiple functions of metabolic regulation. On one hand, prolactin may play an important role in promoting beta-cell proliferation and insulin secretion during pregnancy (5). On the other hand, high prolactin levels may also contribute to insulin resistance, through its effects on dopamine down-regulation (33) and leptin resistance (34) in the central nervous system and the inhibition of lipoprotein lipase activity (35) and adiponectin secretion (36) in adipose tissue. Our finding of a significant and *positive* association of prolactin with insulin and C-peptide levels at weeks 10–14 may reflect a direct effect of prolactin on insulin secretion, or an effect of prolactin on insulin resistance which subsequently triggers increased insulin secretion. Interestingly, at weeks 15–26, prolactin became significantly and *inversely* related to C-peptide levels. It is not clear why the association between prolactin and C-peptide levels changed direction from early to mid-pregnancy. However, samples at the two time-points differ by their fasting status; further, a change in the effect of prolactin is not inconceivable given the dynamic changes in the metabolic (1, 37) and hormonal (2) environments of pregnancy. Roles of prolactin on both insulin resistance and decreased insulin secretion (i.e., inverse association with C-peptide levels) are consistent with our finding of a positive association between prolactin and GDM risk. More broadly, prolactin action is considered essential for metabolic homeostasis during pregnancy and a lack of prolactin receptors resulted in gestational diabetes in animal models (38–40). As placental lactogens also signal through prolactin receptor and have overlapping functions with prolactin (5), they may also be examined in relation to GDM in future studies.

In our study, higher progesterone levels were significantly associated with lower levels of glucose, insulin, and C-peptide at weeks 10–14. Somewhat consistent with this finding, progesterone levels at weeks 10–14 were lower among cases than controls, but the association became non-significant after adjusting for covariates. We are not aware of prior studies of the associations between progesterone and the glucose homeostasis biomarkers during pregnancy. Data on such associations among non-pregnant individuals are generally inconsistent (41–44). As the effects of progesterone on metabolism may vary depending on its concentration (45) and levels of other reproductive hormones (15), it is difficult to compare our findings in pregnant women with those in non-pregnant ones. Future studies among pregnant women are warranted to confirm our findings.

Our study has several strengths. First, it is among the few studies investigating the prospective and longitudinal associations of prolactin and progesterone levels with GDM risk. The prospective hormonal measures before the screening and diagnosis of GDM captured the etiologically relevant window of GDM and established temporal relations between the hormones and GDM risk. The longitudinal measures enabled us to identify the relevant timing of exposure and the trajectories of the hormones throughout pregnancy in relation to GDM risk. Second, this study has the largest sample size among prospective studies. The sample size not only afforded relatively good statistical power but also allowed us to control for potential confounding from selected major risk factors of GDM including pre-pregnancy BMI. Third, the GDM cases were rigorously identified based on independent reviews of well-characterized clinical data, using Carpenter-Coustan criteria. Fourth, the study measured a comprehensive panel of cardiometabolic biomarkers, which provide further insights into potential mechanisms underlying any associations between the hormones and the GDM risk. Lastly, the multi-racial composition of the study sample also contributes to the generalizability of the findings across racial-ethnic groups.

The study has a few potential limitations. First, despite being the largest prospective studies on this topic, our study still had limited power to detect moderate associations. For example, although significant associations were observed between the continuous progesterone levels and glucose metabolism biomarkers at weeks 10–14, the potential association between progesterone levels at weeks 10–14 and GDM risk fail short of significance. Second, due to the practical difficulties of collecting longitudinal fasting samples from pregnant women, only the blood sample collected at weeks 15–26 was fasting, whereas the one at weeks 10–14 was non-fasting. However, this sample collection protocol applied to both cases and controls non-differentially, thus is unlikely to bias the findings. Third, our study is based on pregnant women without major chronic conditions, which may limit the study generalizability. However, there is no obvious known reason that our findings would not apply to the general population of pregnant women of whom the majority are free of major chronic conditions. Lastly, the statistical approaches (e.g., log-transformation, linear mixed-effects models with random intercept) used in comparing the hormonal levels between cases and controls are slightly complex, but they were required by the nature of the data and the study design and necessary for correct analyses of results.

This study found the first prospective evidence of a positive association between prolactin levels in early pregnancy and subsequent risk of GDM. It suggests that higher prolactin levels in early pregnancy may be involved in the pathophysiology of GDM, long before the diagnosis of GDM in the second half of pregnancy. Further research is needed to further elucidate the mechanisms in which prolactin is involved in GDM development.

## Data Availability Statement

The datasets for this article are not currently available for sharing. Requests to access the datasets should be directed to (Cuilin Zhang, zhangcu@mail.nih.gov).

## Ethics Statement

The studies involving human participants were reviewed and approved by NICHD IRB Office. The patients/participants provided their written informed consent to participate in this study.

## Author Contributions

ML analyzed data and wrote the first draft of the manuscript. SR contributed to the conceptualization of the study and revised the manuscript. SH contributed to data interpretation and revised the manuscript. YZ, FT-A, AF, MT, and YS reviewed and revised the manuscript. CZ obtained funding, designed and oversaw the study, and revised the manuscript. All authors interpreted the results, revised the manuscript for important intellectual content, and approved the final version of the manuscript. ML and CZ are the guarantors of this work and, as such, had full access to all the data in the study and take responsibility for the integrity of the data and the accuracy of the data analysis.

### Conflict of Interest

The authors declare that the research was conducted in the absence of any commercial or financial relationships that could be construed as a potential conflict of interest.
