# Supplementary material for: Plasma Prolactin and Progesterone Levels and the Risk of Gestational Diabetes: A Prospective and Longitudinal Study in a Multiracial Cohort
Source: Front Endocrinol (Lausanne). 2020 Feb 27;11:83. doi: 10.3389/fendo.2020.00083 (PMC7058109; doi:10.3389/fendo.2020.00083)
Supplement: Supplementary file 1 [file Table_1.docx]

Supplementary Material

**Supplementary Table 1.** Prolactin and progesterone levels by selected covariates in cases and controls at gestational weeks 10-14 to 15-26, the NICHD Fetal Growth Studies-Singleton Cohort

|  | Cases | | | Controls | | |
| --- | --- | --- | --- | --- | --- | --- |
|  | Median | Mean | SD | Median | Mean | SD |
| Prolactin (ng/mL) | | | | | | |
| Family history of diabetes |  |  |  |  |  |  |
| No | 51.9 | 58.5 | 29.8 | 41.6 | 48.0 | 26.5 |
| Yes | 47.7 | 54.0 | 30.3 | 44.8 | 57.2 | 35.0 |
| Pre-pregnancy BMI, kg/m^2^ |  |  |  |  |  |  |
| < 25 | 52.5 | 62.5 | 35.4 | 39.6 | 46.2 | 24.0 |
| 25-29 | 53.9 | 58.7 | 27.0 | 47.4 | 58.7 | 35.8 |
| >=30 | 45.2 | 49.1 | 25.2 | 42.9 | 49.4 | 29.5 |
| Progesterone (nmol/L) | | | | | | |
| Family history of diabetes |  |  |  |  |  |  |
| No | 106.1 | 113.4 | 39.1 | 127.4 | 126.6 | 31.9 |
| Yes | 112.2 | 122.1 | 39.8 | 121.9 | 119.6 | 39.3 |
| Pre-pregnancy BMI, kg/m^2^ |  |  |  |  |  |  |
| < 25 | 138.7 | 140.0 | 36.7 | 139.9 | 138.2 | 26.4 |
| 25-29 | 101.0 | 107.4 | 38.1 | 121.3 | 120.2 | 33.1 |
| >=30 | 93.5 | 98.0 | 30.2 | 78.6 | 91.3 | 30.1 |

**Supplementary Table 2.** Odds ratios (95% confidence intervals) of GDM associated with prolactin levels (per 10 ng/mL) at gestational weeks 10-14 and 15-26, without and with additional adjustment^a^ for biomarkers of iron status, thyroid function, and insulin-like growth factors (IGF), the NICHD Fetal Growth Studies-Singleton Cohort.

|  | Gestational weeks 10-14 | | Gestational weeks 15-26 | |
| --- | --- | --- | --- | --- |
|  | OR (95% CI) | P-value | OR (95% CI) | P-value |
| *None* | *1.13 (1.03, 1.25)* | *0.02* | *1.08 (1.02, 1.14)* | *0.01* |
| Ferritin | 1.14 (1.03, 1.26) | 0.01 | 1.08 (1.02, 1.14) | 0.01 |
| Hepcidin | 1.13 (1.02, 1.25) | 0.02 | 1.08 (1.02, 1.15) | 0.01 |
| sTFR | 1.13 (1.02, 1.25) | 0.01 | 1.08 (1.02, 1.14) | 0.01 |
| sTFR:Ferritin ratio | 1.13 (1.02, 1.24) | 0.02 | 1.08 (1.02, 1.14) | 0.01 |
| IGF-1 | 1.12 (1.02, 1.24) | 0.02 | 1.07 (1.01, 1.14) | 0.02 |
| IGFBP-2 | 1.11 (1.00, 1.23) | 0.05 | 1.08 (1.02, 1.14) | 0.01 |
| IGFBP-3 | 1.13 (1.03, 1.25) | 0.01 | 1.08 (1.02, 1.14) | 0.01 |
| IGF1-BP3 | 1.12 (1.02, 1.24) | 0.02 | 1.07 (1.01, 1.14) | 0.02 |
| fT3 | 1.14 (1.03, 1.26) | 0.02 | 1.10 (1.03, 1.17) | 0.00 |
| fT4 | 1.14 (1.04, 1.27) | 0.01 | 1.07 (1.01, 1.13) | 0.03 |
| TSH | 1.15 (1.04, 1.27) | 0.01 | 1.08 (1.02, 1.14) | 0.01 |
| fT4:fT3 ratio | 1.15 (1.04, 1.27) | 0.01 | 1.08 (1.02, 1.15) | 0.01 |

^a^All models adjusted for maternal age (years), gestational age (weeks) at blood collection, pre-pregnancy BMI (kg/m^2^), and family history of diabetes (yes, no).

Abbreviations: sTFR – soluble transferrin receptor; IGF – insulin-like growth factor; IGFBP – insulin-like growth factor binding protein; fT3 – free Triiodothyronine; fT4 – free Thyroxine; TSH – thyroid stimulating hormone.

**Supplementary Table1 3.** Longitudinal trajectories of prolactin and progesterone levels from gestational weeks 10-14 to 15-26 for GDM cases and non-GDM controls in the NICHD Fetal Growth Studies-Singleton Cohort

|  | Crude | | Adjusted^a^ | |
| --- | --- | --- | --- | --- |
|  | β (95% CI) | *P*-value | β (95% CI) | *P*-value |
| Prolactin, ng/mL |  |  |  |  |
| GDM | 0.18 (0.01, 0.35) | 0.04 | 0.22 (0.05, 0.40) | 0.01 |
| Visit | 0.91 (0.79, 1.03) | < 0.001 | 0.91 (0.79, 1.03) | < 0.001 |
| GDM*Visit | -0.02 (-0.23, 0.20) | 0.87 | -0.02 (-0.23, 0.19) | 0.87 |
| Progesterone, nmol/L |  |  |  |  |
| GDM | -0.09 (-0.17, -0.01) | 0.03 | -0.04 (-0.11, 0.04) | 0.33 |
| Visit | 0.31 (0.27, 0.36) | < 0.001 | 0.31 (0.27, 0.36) | < 0.001 |
| GDM*Visit | 0.06 (-0.02, 0.14) | 0.14 | 0.06 (0.14, -0.02) | 0.14 |

^a^Adjusted for maternal age (years), pre-pregnancy BMI (kg/m^2^) and family history of diabetes (yes, no).

β coefficient for “GDM” represents the log-transformed difference of the hormonal levels between GDM cases and non-GDM controls at gestational weeks 10-14;

β coefficient for “Visit” represents the log-transformed longitudinal change of hormonal levels from gestational weeks 10-14 to 15-26 among non-GDM controls;

β coefficient for “GDM*Visit” represents the log-transformed difference in longitudinal change of hormonal levels from gestational weeks 10-14 to 15-26 between GDM cases and non-GDM controls.
